# Prevalence and severity of nomophobia among nurses: A systematic review and meta-analysis

**DOI:** 10.17533/udea.iee.v42n3e05

**Published:** 2024-10-19

**Authors:** Shiv Kumar Mudgal, Suresh Kumar Sharma, Rakhi Gaur, Maneesh Sharma, Latha T, Vipin Patidar

**Affiliations:** 1 .Ph.D. Associate Professor. Email: shiv.nur@aiimsdeoghar.edu.in https://orcid.org/0000-0002-8062-0589 All India Institute Of Medical Sciences India shiv.nur@aiimsdeoghar.edu.in; 2 . Ph.D, Professor & Principal. Email: sk.aiims17@gmail.com https://orcid.org/0000-0003-1214-8865 All India Institute Of Medical Sciences India sk.aiims17@gmail.com; 3 . Ph.D, Assistant Professor. Email: rakhi.nur@aiimsdeoghar.edu.in https://orcid.org/0000-0003-0835-4383 All India Institute Of Medical Sciences India rakhi.nur@aiimsdeoghar.edu.in; 4 . M.Sc. Assistant Professor. Email: maneesh.nur@aiimsrishikesh.edu.in https://orcid.org/0000-0003-0204-2258 All India Institute Of Medical Sciences India maneesh.nur@aiimsrishikesh.edu.in; 5 . Ph.D, Associate Professor. Email: latha.nursing@aiimskalyani.edu.in https://orcid.org/0000-0003-0856-4380 All India Institute Of Medical Sciences India latha.nursing@aiimskalyani.edu.in; 6 . M.Sc. Tutor/clinical Instructor. India. Email: vipin.nur@aiimsdeoghar.edu.in. Corresponding author. https://orcid.org/0000-0003-4595-9859 All India Institute Of Medical Sciences India vipin.nur@aiimsdeoghar.edu.in; 7 . College of Nursing, All India Institute of Medical Sciences, Deoghar, Jharkhand, India. All India Institute Of Medical Sciences College of Nursing All India Institute of Medical Sciences Deoghar Jharkhand India; 8 . College of Nursing, All India Institute of Medical Sciences, Jodhpur, Rajasthan, India All India Institute Of Medical Sciences College of Nursing All India Institute of Medical Sciences Jodhpur Rajasthan India; 9 . College of Nursing, All India Institute of Medical Sciences, Rishikesh, Uttarakhand, India All India Institute Of Medical Sciences College of Nursing All India Institute of Medical Sciences Rishikesh Uttarakhand India; 10 . College of Nursing, All India Institute of Medical Sciences, Kalyani, West Bengal, India. All India Institute Of Medical Sciences College of Nursing All India Institute of Medical Sciences Kalyani West India

**Keywords:** meta-analysis, nurse, smartphone, systematic review, metaanálisis, enfermeras y enfermeros, teléfono inteligente, revisión sistemática, metanálise, enfermeiras e enfermeiros, smartphone, revisão sistemática.

## Abstract

**Objectives.:**

To determine the prevalence and severity of nomophobia (dread of not having a smartphone) among nurses.

**Methods.:**

A systematic search was carried out across different electronic databases, including Medline (PubMed), SCOPUS Embase, CINAHL, EBSCO, and Google Scholar, until March 2024. The meta-analysis included studies that reported the prevalence of nomophobia in nurses and used the Nomophobia Questionnaire (NMP-Q). Two independent reviewers identified the studies, extracted the data, and assessed the risk of bias using Joanna Briggs Institute Critical Appraisal Tool. PROSPERO register number CRD42024512079.

**Results.:**

A total 10 studies (4 in Italy and 6 in Turkey) with 3086 individuals were found to meet the inclusion criteria for the systematic review. However, data could not be retrieved for one research, thus nine studies being included in the meta-analysis. The Overall Prevalence of nomophobia was 68.15% (95% CI: 57.49%-78.81%; I² = 99%). The prevalence of mild nomophobia was reported to be 43% (95% CI, 24%-65%; I^2^ = 99%), moderate nomophobia was 31% (95% CI, 17%-50%; I^2^ = 99%), and severe nomophobia was 7% (95% CI, 2%-25%; I^2^= 95%). Country-specific analysis revealed that Turkish nurses had a greater level of nomophobia than their Italian nurses.

**Conclusion.:**

Nurses have a high prevalence of mild to moderate nomophobia which emphasizes the need of preventative initiatives and tailored intervention for nurses in health care organizations.

## Resumen

**Objetivo.:**

Determinar la prevalencia y severidad de la nomofobia (temor a no disponer de un teléfono inteligente) entre las enfermeras.

**Métodos.:**

Se realizó una búsqueda sistemática en diferentes bases de datos electrónicas, incluyendo Medline (PubMed), SCOPUS Embase, CINAHL, EBSCO y Google Scholar, hasta marzo de 2024. El metaanálisis incluyó estudios que informaron sobre la prevalencia de la nomofobia en enfermeras y que utilizaron el Nomophobia Questionnaire (NMP-Q). Dos revisores independientes identificaron los estudios, extrajeron los datos y evaluaron el riesgo de sesgo mediante la Herramienta de Evaluación Crítica del Instituto Joanna Briggs. Registro PROSPERO número CRD42024512079**.**

**Resultados.:**

Se revisaron un total de 10 estudios (4 en Italia y 6 en Turquía) con un total de 3086 individuos, de los cuales nueve se incluyeron en el metaanálisis. La prevalencia de nomofobia fue: global: 68.15% (95% CI: 57.49%-78.81%; I² = 99%), leve: 43% (IC 95%, 24%-65%; I^2^ = 99%), moderada: 31% (IC 95%, 17%-50%; I^2^ = 99%) y severa: del 7% (IC 95%, 2%-25%; I^2^= 95%). El análisis por países reveló que las enfermeras turcas tenían un mayor nivel de nomofobia que las italianas.

**Conclusión.:**

Las enfermeras tienen una alta prevalencia de nomofobia de leve a moderada, lo que enfatiza la necesidad de iniciativas preventivas y de intervención para las enfermeras en las organizaciones de salud.

## Resumo

**Objetivo.:**

Determinar a prevalência e gravidade da nomofobia (medo de não ter smartphone) entre enfermeiros.

**Métodos.:**

Foi realizada uma pesquisa sistemática em diferentes bases de dados eletrônicas, incluindo Medline (PubMed), SCOPUS Embase, CINAHL, EBSCO e Google Scholar, até março de 2024. A meta-análise incluiu estudos que relataram a prevalência de nomofobia em enfermeiros e que utilizaram o Questionário de Nomofobia (NMP-Q). Dois revisores independentes identificaram estudos, extraíram dados e avaliaram o risco de viés usando a ferramenta de avaliação crítica do Joanna Briggs Institute. Registro PROSPERO número CRD42024512079**.**

**Resultados.:**

Foram revisados 10 estudos (4 na Itália e 6 na Turquia) com um total de 3.086 indivíduos, dos quais nove foram incluídos na meta-análise. A prevalência de nomofobia foi: geral: 68.15% (IC 95%: 57.49%-78.81%; I² = 99%), leve: 43% (IC 95%, 24%-65%; I^2^ = 99%), moderada: 31% (IC 95%, 17%-50%; I^2^ = 99%) e grave: 7% (IC 95%, 2%-25%; I ^2^ = 95%). A análise por país revelou que os enfermeiros turcos tinham um nível de nomofobia mais elevado do que os enfermeiros italianos.

**Conclusão.:**

Os enfermeiros apresentam alta prevalência de nomofobia leve a moderada, enfatizando a necessidade de iniciativas preventivas e de intervenção para os enfermeiros nas organizações de saúde.

## Introduction

Data and communication technology have become an indispensable part of our modern civilization.^1^ While its integration has improved and streamlined everyday activities, providing countless advantages to individuals^2^, it has also resulted in concerns related to addiction and an outbreak of issues related to mental health.^3^ The pervasive and persuasive nature of smartphones has fostered negative habits among young people, akin to compulsive behaviours such as incessantly checking the phone for missed messages or calls, verifying the availability of a web connection, keeping the phone switched on 24/7, never leaving home without the mobile device, and using the phone even during conversations, thereby disregarding the other person (a behaviour known as "phubbing").^4^ Furthermore, people may suffer "ringxiety," a phrase derived from "ring" and "anxiety," in which they falsely assume they have heard the phone ring.^5^ These symptoms together appear as "Nomophobia," which is the dread of being disconnected or unable to utilize a mobile phone.^6^ Other characteristics includes feelings of worry, emotional instability, hostility, discomfort, and difficulty in focus.^7^

The growing usage of mobile devices in the workplace has resulted in less time spent on tasks and more work interruptions. This has caused a shift in the nature of many employments, including those in the healthcare industry.^8^ Nurses with high degrees of nomophobia frequently check their mobile device alerts^9^ and this practice has a negative impact on many aspects of their lives, including sleep quality, eating habits, overall health, physical activity, attention span, and importantly their health care practices.^10^ Because of nomophobia, nurses working in specialized units such as intensive care services, trauma and emergency, cardiac unit etc. may unknowingly overlook their caring obligations, resulting in medical errors. These mistakes can lengthen patients' hospitalizations, increase the cost of care per patient, and perhaps result in debilitating repercussions or even patient death.^11^^,^^12^ There were some studies ^13^^,^^14^ which reported that majority of nurse had mild level of nomophobia while other studies ^15^^,^^16^ reported majority of nurse had moderate to severe level of nomophobia, emphasizes the complexity of the issue. Given this variance, it is critical to derive conclusions using a systematic review and meta-analysis method. So, our study aims to integrate current data on the prevalence of nomophobia among nurses in response to the growing challenge given by an expanding digital culture and the scarcity of research. Furthermore, we intend to identify the severity levels among nurses.

## Methods

The systematic review adhered to the Preferred Reporting Items for Systematic Reviews and Meta-Analyses (PRISMA Guidelines)^17^ and Meta-analysis of Observational Studies in Epidemiology (MOOSE) criteria^18^ (attached in Supplementary file S1 and S2 respectively). The study protocol was registered with PROSPERO under the registration number CRD42024512079.

Information Resources and the Search Equation. A systematic search was performed until March 2024, using five databases: PubMed, Scopus, Embase, CINAHL, and Google Scholar. The search strategy included MeSH/All term descriptors and various terms such as "Nomophobia," "No mobile phobia," "No smartphone phobia," "No mobile-phone phobia," "No smart-phone phobia," "fear of being without a mobile phone," "nomofobia," "fear of missing out cell phone," "fear of being without a smartphone," as well as terms related to nurses such as 'nurse,' 'nurses,' 'registered nurse,' 'RN,' 'Nursing officer,’ and 'professional nurse.' Supplementary file S3 includes detailed search algorithms for each database. Furthermore, the reference lists from the selected researches were screened to find other relevant researches. The identified references were imported into Mendeley, and duplicates were deleted. Following that, two researchers independently looked into publications based on titles and abstracts to find possibly relevant items for inclusion. Selected research papers underwent full-text screening, which was additionally carried out separately by two authors. Any disagreements were handled through a conversation with a third author to reach an agreement on the final conclusion.

Study selection. Following the elimination of duplicate data, two reviewers independently evaluated the remaining records' titles and abstracts to identify possibly relevant research. The entire texts were then collected and evaluated separately by two reviewers. Studies were considered eligible if they met the following requirements: The inclusion criteria were as follows: (a) original, peer-reviewed research published in English; (b) a focus on nurses’ cohorts; and (c) a review of Nomophobia-related features among nurses. Studies with fewer than 50 participants, duplicate cohorts, and those lacking sufficient individual-level data on nurses or unavailable through all useful approaches were excluded, as were case-control studies, case reports, editorials, commentaries, clinical practice guidelines, opinions, and reviews also.

Codification of the findings. Two reviewers gathered data separately using a Microsoft Excel file with established data extraction parameters. The retrieved data contained: Study features include the title, journal, author(s), publication year, country, study methodology, nomophobia measuring tool, and risk of bias assessment; (ii) At the study level, participant information includes age (mean, standard deviation, or range) and gender (male/female ratio); (iii) Key data: sample size, nomophobia features such as prevalence, mean and standard deviation of nomophobia scores, nomophobia categories, and quality of evidence were all investigated. A third reviewer settled disagreements about inclusion or exclusion criteria. If necessary, information was not found in the research, attempts were made to contact the authors via email.

Risk of Bias. The methodological rigor of prevalence studies was rated separately by two researchers using the Joanna Briggs Institute Critical Appraisal Tool.^19^ A third author rectified the discrepancies. This evaluation tool consists of nine items with response possibilities of "yes," "no," or "unclear" if insufficient data prohibited a definitive conclusion concerning the issue. Each conforming item was given one point, whereas non-compliance or ambiguous replies earned zero points. Methodological quality was measured using the total score, with values of 0-3 indicating low quality, 4-6 moderate quality, and 7-9 excellent quality in the prevalence analysis of bias risk.

Statistical Analysis. We used a meta-analysis to determine the overall prevalence and severity of nomophobia among nurses. A random-effects model was used, with 95% confidence intervals (CIs). The Cochrane Q statistic and I² test were used to examine heterogeneity and its origins. Subgroup analyses were carried out according to country. All statistical analyses were carried out using R software (version 4.2.3). The "metaprop" and "metamean" functions in R (version 4.2.3) were used to calculate the pooled prevalence and general mean of nomophobia among nurses, respectively.

Subgroup Analysis. A country-specific subgroup analysis was performed to determine the prevalence of nomophobia among nurses.

**Figure 1:**
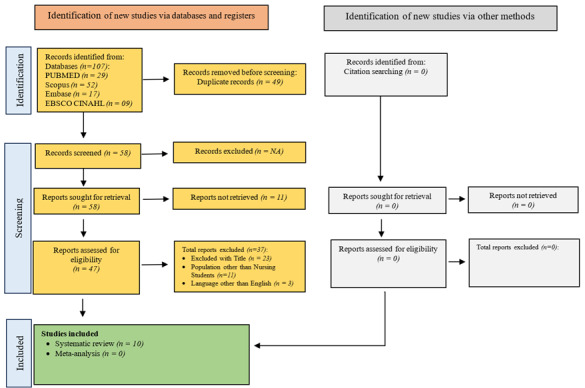
PRISMA flow Diagram

Publication Bias. The primary outcome was not evaluated for publication bias due to the small number (<10) of research articles included in the systematic review and meta-analysis.

## Results

Search results. The initial database search yielded 107 research articles. After deleting duplicate entries, we identified 58 unique researches. Following a full-text evaluation of 47 papers, 10 satisfied the inclusion criteria. Furthermore, no other studies were found by reference filtering. As a result, the systematic review comprised ten research articles (see Figure 1).

Baseline characteristics of included studies. Ten studies with a total of 3,086 individuals were taken into consideration (Table 1). In terms of nations represented, six studies were done in Turkey^11^^,^^16^^,^^20^^-^^23^ and four in Italy^12^^-^^15^ Participants' average ages ranged from 28.4 to 41.2 years. In terms of nomophobia classification, nine studies^11^^,^^13^^-^^16^^,^^20^^-^^23^ used mean and standard deviation to assess overall nomophobia levels, while five studies^11^^,^^13^^-^^16^ divided nomophobia into four groups (absent: 20; mild: 21-59; moderate: 60-99; severe: 100-140), and one study^12^ used statistical methods to categorize nomophobia (NMP-Q) into a five-point scale ranging from 1-5, which was not included in the meta-analysis.

**Table 1:** Summary table of studies included in the systematic review on prevalence and severity of nomophobia among nurses

Author & year	Country	Design	Scale	Participants characteristics			Findings		Evidence/ degree of recommendation
				N	Male	Female	Age (mean ± SD)		
Bülbüloğlu *et al.* (2019)	Turkey	The descriptive and cross-sectional design	NMP-Q	304	109	205	Not reported	The Nomophobia total score was 60.77 ± 15.09.	Moderate quality
Cetin *et al.* (2019)	Turkey	The descriptive and correlational research	NMP-Q	284	66	218	29.50±5.76	The Nomophobia total score was 90.09 ± 28.47.	High quality
Demirel *et a*l. (2022)	Turkey	The descriptive and relationship-seeking design	NMP-Q	285	50	235	29.67±7.62	The Nomophobia total score was 77.65 ± 25.76.	High quality
Frassini *et al*. (2021)	Italy	A cross-sectional quantitative descriptive study	NMP-Q	139	34	105	41.2 ± 10.2	The Nomophobia total score was 79.3 ± 30.7. **Nomophobia categories** Mild 25.2% (n=35) Moderate 48.2% (n=67) Severe 25.2% (n=35)	Moderate quality
Hoşgör et al. (2021)	Turkey	The descriptive study	NMP-Q	178	18	160	30.54 ± 7.30	The Nomophobia total score was 50.8 ± 17.26. **Nomophobia categories** Mild 37.6% (n=67) Moderate 25.2% (n= 45) Severe 5.1% (n=9)	Moderate quality
Kapikiran *et al.* (2023)	Turkey	The descriptive and cross-sectional design	NMP-Q	186	38	148	33.37 ± 7.15	The Nomophobia total score was 66.64 ± 25.36.	High quality
Lupo *et al.* (2020)	Italy	Transversal and observational multicentre study	NMP-Q	539	144	395	33.8 ±13.11	The Nomophobia total score was 50.34 ± 29.032. **Nomophobia categories** Mild nomophobia 66.2% (n=347) Moderate nomophobia 21% (n=110) Severe nomophobia 6.9% (n=36)	Moderate quality
Marletta *et al.* (2021)	Italy	Observational and descriptive study	NMP-Q	72	Not reporter	Not reported	Not reported	The Nomophobia total score was 2.67 ± 1.15.	Moderate quality
Uguz *et al.* (2021)	Turkey	The descriptive, cross‐sectional, and correlational study.	NMP-Q	669	115	554	28.40 (6.54)	The Nomophobia total score was 78.17 ± 22.58. **Nomophobia categories** Mild 20.9% (n=140) Moderate 59.2% (n=396) Severe nomophobia 19.4% (n=130)	Moderate quality
Vitale *et al.* (2023)	Italy	The cross‐sectional, and analytical	NMP-Q	430	105	325	37 ± 12	The Nomophobia total score was 60.03 ± 26.60. **Nomophobia categories** Mild 71.6% (n=308) Moderate 13.5% (n=58) No respondents record severe nomophobia levels.	Moderate quality

NMP‐Q- Nomophobia Questionnaire

**Figure 2:**
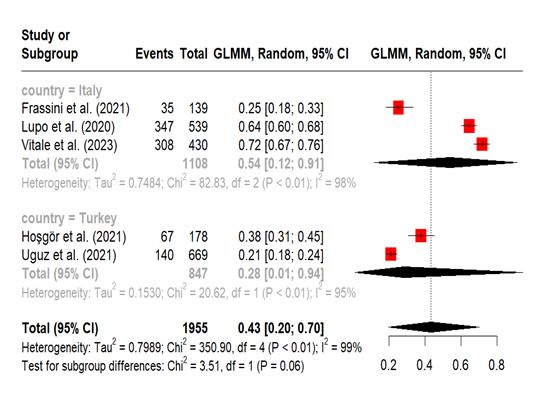
Forest plot, Mild Nomophobia in Nurse’s (Meta-Analytical Estimation)

**Figure 3:**
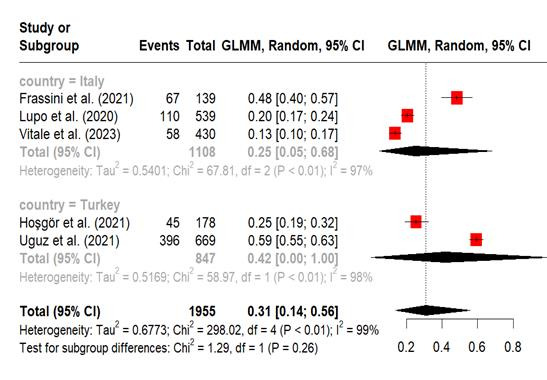
Forest plot, Moderate Nomophobia in Nurses (Meta-Analytical Estimation)

**Figure 4:**
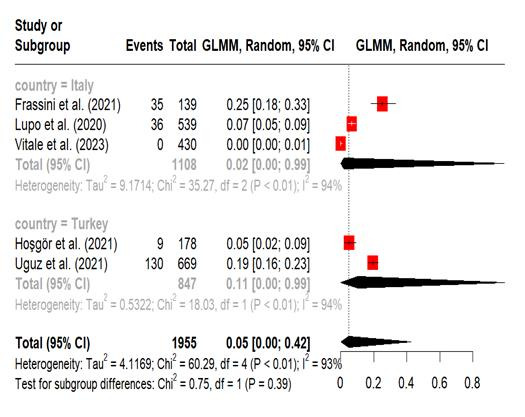
Forest plot, Severe Nomophobia in Nurses (Meta-Analytical Estimation).

Pooled prevalence of nomophobia in nurses. A meta-analysis was carried out on five studies that reported the prevalence of nomophobia among nurses, using established cut-off points to classify the condition as mild, moderate, and severe.^11^^,^^13^^-^^16^ The prevalence of mild nomophobia was 43% (95% CI, 20%-70%; I^2^ = 99%). The subgroup analysis by country indicated that the pooled prevalence of mild nomophobia among nurses residing in Italy (54% [95% CI 12%-91%; I^2^ = 98%, P <0.01]) was greater than that among nurses living in Turkey. The pooled prevalence of moderate nomophobia was 31% (95% CI, 14%-56%; I^2^ = 99%). Subgroup analysis revealed that nurses in Turkey had a greater moderate level of nomophobia (41% [95% CI, 0%-100%; I^2^ = 98%]) than those in Italy. The prevalence of severe nomophobia was 5% (95% CI, 0%-42%; I^2^ = 93%). Nurses in Turkey had a greater prevalence (11% [95% CI, 0%-99%; I^2^ = 94%]) than in Italy. The meta-analysis repeatedly showed high heterogeneity (Figures 2, 3 and 4)

**Figure 5:**
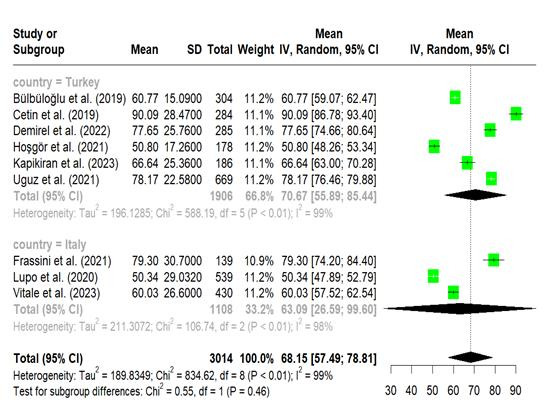
Forest plot, Overall Nomophobia in Nurses (Meta-Analytical Estimation)

Overall mean of nomophobia in nurses. A meta-analysis shown in figures 5 was performed on nine studies that reported the mean nomophobia score in nurses.^11^^,^^13^^-^^16^^,^^20^^-^^23^ The average score for nomophobia was 68.15 (95% CI, 57.49-78.81; I^2^ = 99%). Subgroup analysis based on nations revealed that the Turkish nurses had a higher mean score of nomophobia (70.67 [95% CI -55.89-85.44; I^2^ = 99%, P <0.01]) than Italian nurses (63.09 [95% CI - 26.59-99.60; I^2^ = 98%, P <0.01].

Risk of Bias. The JBI scores in the included reports ranged between 5 to 7. Seven of the 10 studies have been categorized as moderate quality, with three categorized as high quality. There were no reports categorized as low quality. Supplementary file S4 offers complete scores for all included studies.

Publication bias. The primary outcome was not evaluated for publication bias due to the small number (<10) of research articles included in the systematic review and meta-analysis. This limited numbers of research articles hindered the ability to adequately assess publication bias.

## Discussion

Smartphones provide a variety of features to meet users' everyday requirements, including communication, scheduling, online surfing, social networking, and entertainment.^24^^,^^25^ Despite the benefits, excessive smartphone usage can cause psychological distress, especially among youths. This reliance on mobile technology has raised concerns about its impact on mental and physical health, with severe cases of nomophobia, which is defined as fear and anxiety when separated from technology, being linked to an increased risk of depression, anxiety, stress, musculoskeletal issues, and even the vehicular accidents.^26^^,^^27^ In today's smartphone-dependent world, Nomophobia, or the dread of being without a mobile device, causes people to keep their phones nearby at all times, even when sleeping, and often carry multiple devices or chargers as a backup. This fear has been linked to a variety of mental health problems, including stress, sleeplessness, anxiety, depression, and personality disorders, as well as low self-esteem, all of which have an influence on cognitive and motor skills.^(1, 26)^ To the best of the authors' knowledge, this is one of the first attempts to perform a systematic review and meta-analysis to determine the prevalence of nomophobia among nurses. The purpose of this research aims to determine the prevalence of nomophobia among nurses and investigate its ramifications in order to inform initiatives targeted at encouraging safe smartphone usage among prospective nurses and other healthcare practitioners. 

Though, we could not retrieve meta-analysis on this topic to discuss our findings therefore we compared the finding of the present study with other similar type of studies. The studies featured, predominantly from 2020, used quantitative and cross-sectional methodologies, mostly in an exploratory stage. We found a significant severity of nomophobia among nurses, with 68.15% feeling it to some extent, indicating its pervasive impact. Turkey was identified as the major source of research throughout the country-specific evaluation. The symptoms intensity varied, with 43% reporting mild symptoms, 31% moderate, and 7% severe, in line with previous study findings.^9^^,^^26^

The remarkable heterogeneity among the researches is an important note in the findings. This variance may be due to a variety of factors, including differences in research design, geographical contexts, and cultural inequalities amongst the study populations. Notably, differences in smartphone usage patterns, technological availability, and social attitudes toward smartphone may impact the prevalence and severity of nomophobia in different nations. Particularly, there were geographic variations, with Turkish nurses showing more severity of nomophobia than their Italian counterparts. These discrepancies highlight the need of considering sociocultural influences when assessing nomophobia. Furthermore, the majority of research were done during and following COVID-19, indicating a probable association between increased smartphone usage and nomophobia.

It is of the utmost importance for healthcare organizations to emphasize the development of policies and resources aimed at encouraging appropriate smartphone use among nurses. This includes initiatives such as educational programs to raise awareness about the risks associated with excessive smartphone use, the development of clear guidelines governing smartphone use in clinical settings, and the provision of supportive services to individuals struggling with technology addiction. By implementing these methods, institutions may actively reduce the negative impacts of nomophobia while also promoting general well-being among nurses. These measures are essential for ensuring a healthy balance between technology integration and professional obligations in the healthcare context.^27^^,^^28^ The findings of this systematic review highlight the significant prevalence and severity of nomophobia among nurses, emphasizing the need for additional research and tailored interventions in this area. A better understanding of the factors that contribute to nomophobia and its consequences in health care setting enables Nurse manager to effectively implement preventive measures.

Our study's key strength is that it does subgroup analysis based on nation, providing useful insights into the variations in nomophobia prevalence and severity among nurses across different geographic locations. This approach broadens our awareness of the importance of social factors in explaining technological behaviours. The result of this systematic review and meta-analysis will be considered with following limitation. 1) Despite efforts to search different databases, only publications from two countries were found, indicating a lack of regional representation. 2) Our review only included research published in English. 3) There is significant variation despite the consistent use of NMP-Q cut-off points for nomophobia severity categorization, with one research excluded due to non-standardized cut-offs.

Conclusion. The systematic review highlights a significant prevalence of nomophobia among nurses, with varying degrees of severity across two nations. This diversity suggests that a universal solution may not suffice, highlighting the need for tailored measures to address nomophobia effectively among nurses, considering specific circumstances and demographics. 
